# Case of a Child with Two Heads

**Published:** 1840

**Authors:** 


*Case of a child with two heads.*—On the seventh day of October, in the year 1836, I was called to visit a lady by the name of McK., who was at that time in labor. On examination the umbilical cord had protruded into the vagina—the pulsation was then very strong. In fifteen or twenty minutes after, another examination was made when the pulsation had ceased, and and the foot and leg advanced. I then made exertion to bring the head forward—to my astonishment, I found the neck of a child with a perfect finger adjoined to the extreme end of it. This being brought down, I discovered the head was large and would be attended with great difficulty in delivering, the woman being very small, with a narrow pelvis. I, however, succeeded in a few minutes in delivering her of a child with two heads, adjoined together at the crowns —the faces reverted, the back of the one coming to the forehead of the other—each precisely alike—the one a perfect child, the other a perfect head and neck, and a perfect finger of common size. The heads measured fifteen and a half inches around, where they were joined, and ten inches long. The mother did extremely well.—*Ib.*

---

*Case of twins in which one had been long dead.*—The following evening I was called to visit a lady, by name J., who was in labor. She had been in bad health for several months. I delivered her of a son, healthy looking child, who continues so. I also delivered her of another child which had been dead so long that its joints were separated. The whole body presented a mass of corruption—the skin was not broken.

